# Mobile Phone Use and The Risk of Headache: A Systematic Review and Meta-analysis of Cross-sectional Studies

**DOI:** 10.1038/s41598-017-12802-9

**Published:** 2017-10-03

**Authors:** Jing Wang, Hui Su, Wei Xie, Shengyuan Yu

**Affiliations:** 1 Department of Neurology, Chinese PLA General Hospital, Beijing, 100853 PR China; 2 School of Medicine, Nankai University, Tianjin, 300071 PR China

## Abstract

Headache is increasingly being reported as a detrimental effect of mobile phone (MP) use. However, studies aimed to investigate the association between MP use and headache yielded conflicting results. To assess the consistency of the data on the topic, we performed a systematic review and meta-analysis of the available cross-sectional studies. Published literature from PubMed and other databases were retrieved and screened, and 7 cross-sectional studies were finally included in this meta-analysis. The pooled odds ratio (OR) and 95% confidence interval (CI) were calculated. We found that the risk of headache was increased by 38% in MP user compared with non-MP user (OR, 1.38; 95% CI, 1.18–1.61, *p* < 0.001). Among MP users, the risk of headache was also increased in those who had longer daily call duration (2–15 min vs. <2 min: OR, 1.62; 95% CI, 1.34–1.98, *p* < 0.001; >15 min vs. <2 min: OR, 2.50; 95% CI, 1.76–3.54, *p* < 0.001) and higher daily call frequency (2–4 calls vs. <2 calls: OR, 1.37; 95% CI, 1.07–1.76, *p* < 0.001; >4 calls vs. <2 calls: OR, 2.52; 95% CI, 1.78–3.58, *p* < 0.001). Our data indicate that MP use is significantly associated with headache, further epidemiologic and experimental studies are required to affirm and understand this association.

## Introduction

The use of mobile phones (MP) has significantly increased globally since the 1990s^1^, especially in the last decade. During the last decade, functions other than communication have been integrated into the MP, such as email/Internet access and various forms of entertainment such as videos, music, or games. As the phone becomes more like a personal computer, public concerns about potential detrimental effects on human health from electromagnetic fields emitted by MPs have been raised but laid to rest by science. Even so, minor side effects such as headache, sleep disturbance, lack of concentration, impairment of short-term memory, dizziness, tinnitus, fatigue, and benign warming of the ear have been reported^2–8^.

Headache is common pain syndrome that is reportedly increasing^9^. Headache has been loosely tied to excessive MP use^10–12^, but studies have produced conflicting results. Likely this can be explained by small study populations that were not of sufficient power to indicate benefit or harm. Thus, to gain a better understanding of the relationship between MP use and headache, we performed a quantitative meta-analysis of cross-sectional studies to appraise the association between MP use and headache. This is, to our knowledge, the first systematic review and meta-analysis to quantify the influence of MP use on headache risk.

## Methods

### Search Strategy

The study was performed according to the recommendation of the Preferred Reporting Items for Systematic Reviews and Meta-analysis (PRISMA) guidelines^13^. The literature search was performed in May 2017. All studies, in any language, published between January1, 1990, and May1, 2017, were included. The time frame was selected to reflect the relatively extensive use of mobile phone. PubMed, EMbase and Cochrane databases were systematically searched for relevant studies using search terms: ((mobile phone) OR (cell phone) OR (cellular phone)) AND ((headache) OR (health effect) OR (health symptom) OR (subjective symptom)). The gray literature was searched using the OpenGrey online database. Additionally, we conducted a manual search of references of the included studies and reviews to find more relevant studies. The literature search was conducted independently by two reviewers (Wang and Su).

### Inclusion and exclusion criteria

Studies investigating the association between MP use and headache were evaluated. Search results from the three databases were first imported into Endnote X7 (Thompson Reuter, CA) to remove duplicates, and then two reviewers (Wang and Su) independently screened the remaining references using predetermined inclusion criteria, which were: (1) cross-sectional studies to evaluate the association between MP use and headache; (2) no restriction on language, publication area, and age of the study population; (3) odds ratio (OR) was reported to assess the impact of MP use on headache; and (4) multivariate logistic regression was used to adjust for confounders when OR and 95% confidence intervals (CI) were calculated. Studies were excluded if they were abstracts, reviews, comments, or conference papers or animal studies. If more than one article reported data from one study, the most recent and complete article was included. Discrepancies in screening the articles for the eligibility were discussed between two reviewers (Wang and Su) to reach a consensus. Consultation from supervisor (Yu) was acquired if necessary.

### Data extraction and quality assessment

Two reviewers (Wang and Su) independently extracted and summarized the data of the included studies. The following information was extracted from each included study: first author’s name, publication year, study design, study population, patient numbers, age of study population, gender, exposure source, study groups, outcome assessment and confounders adjusted in the statistical analysis. Information was examined and adjudicated by an additional reviewer (Xie), and discrepancies were resolved by consensus with supervisor (Yu), who referred to the original articles.

The Newcastle-Ottawa Scale (NOS) was used to evaluate the quality of each included study^14^ and this includes eight assessment items for quality appraisal including ‘selection’, ‘comparability’ and ‘outcome’. According to the NOS score standard, cross-sectional studies could be classified as low-quality (scores of 0–4), moderate-quality (scores of 5–6) and high-quality (scores ≥7).

### Statistical analysis

The adjusted OR and corresponding 95% CIs were extracted from each study and used to assess an association between MP use and headache. A chi-square test and I-squared (I^2^) statistic were used to evaluate heterogeneity among included studies. Statistical heterogeneity was considered significant when *p* < 0.10 for χ^2^ test or I^2^ > 50%^15^. If there was heterogeneity among studies, random-effects model was applied to calculate the summary OR, otherwise, the fixed-effects model was used. Visual inspection of the funnel plot was used to confirm publication bias. Egger’s regression test^16^ and Begg’s test^17^ were used to statistically assess publication bias and we performed a sensitivity analysis by excluding one study each time and rerunning the analysis to verify the robustness of the overall results (*p* < 0.05 was considered statistically significant). All analysis was performed using Stata release 11(Stata Corp, College Station, TX).

## Results

### Literature search and Study characteristics

**Figure 1:**
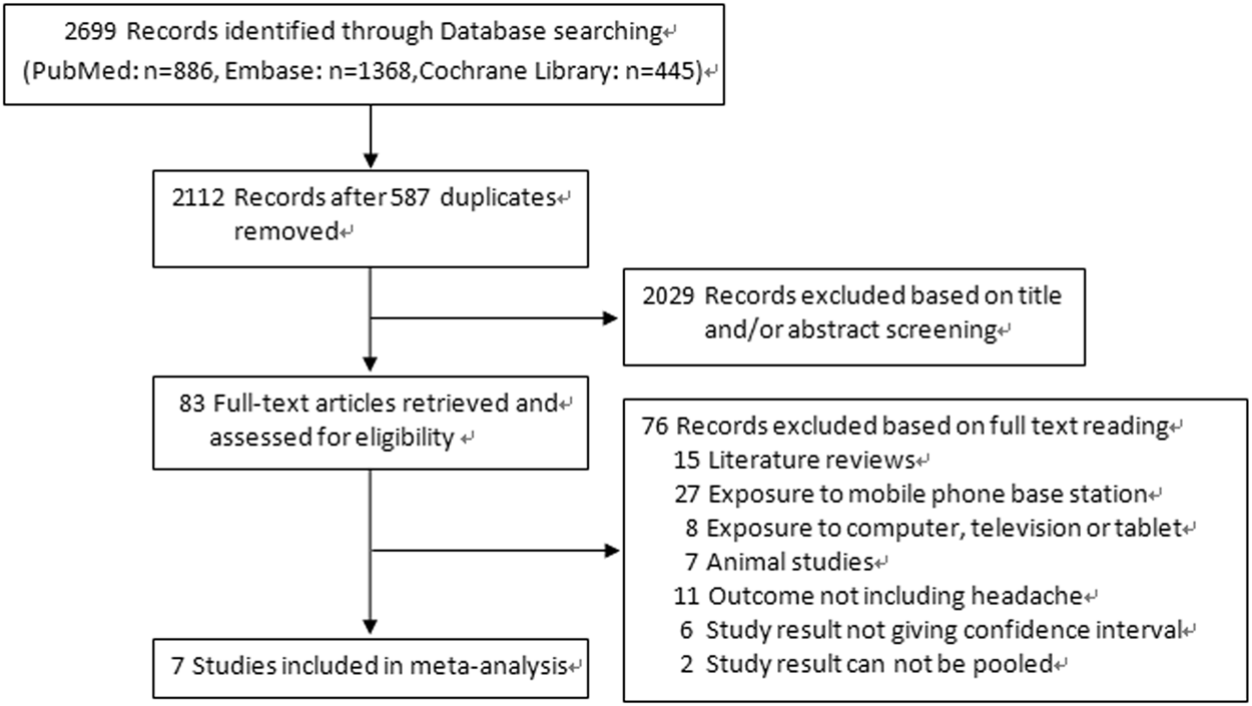
Flow diagram of literature search and study selection.

A total of 2,699 articles were identified through literature searching in PubMed, EMbase and Cochrane databases. The studies assessed are described in Fig. 1. After removing duplicate articles and initial screening based on titles and abstract reading, 83 articles remained for full-text reading. Two independent reviewers performed the full-text reading and finally 7 studies^18–24^ met our inclusion criteria. Of the 76 articles excluded by full-text reading, 15 were review articles, 27 reported headache due to living near MP base station, 8 reported headache due to computer, television or tablet use, 11 reported health outcomes not including headache, 7 were animal studies. There were 8 studies^25–32^ investigated the association between mobile phone use and headache, but not fulfilled the inclusion criteria. Among them, 6 did not give OR and corresponding 95%CI for association between MP use and headache and 2 study results cannot be pooled.

Seven cross-sectional studies involving 21,505 study subjects were included in this meta-analysis. Four studies were conducted in Asia^18–20,24^ and three studies were conducted in Europe^21–23^.Five studies^18–21,24^ assessed the association between MP use or not and headache, two studies^22,23^ assessed the association between MP calling time and headache, one study^23^ assessed the association between MP calling frequency and headache. The main characteristics of the included studies are shown in Supplementary Table S1. Main characteristics of the 8 excluded studies are shown in Supplementary Table S2.

### Quality assessment

The quality of included studies ranged from moderate to high as illustrated in Supplementary Table S1. Specific assessments of included studies are shown in Supplementary Table S3.

### Meta-analysis of MP use and headache risk

#### MP versus non-MP user

**Figure 2:**
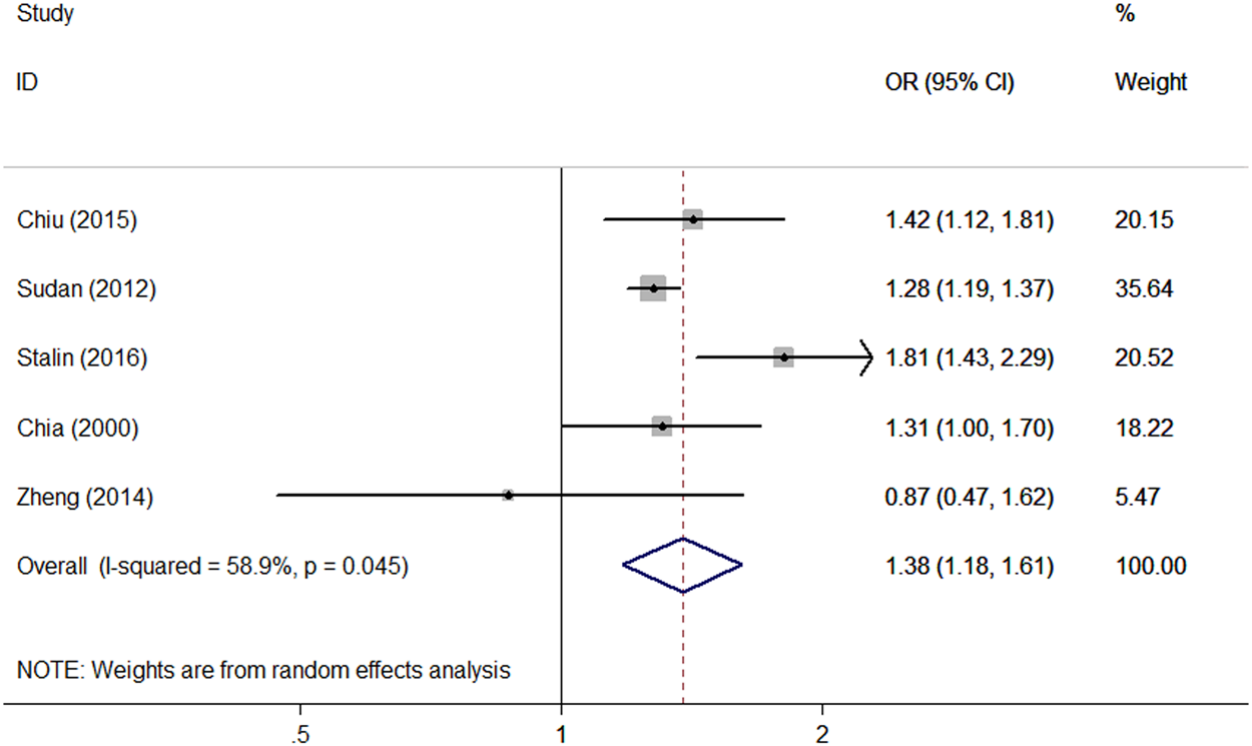
Forest plot of the association between MP use or not and headache.

**Figure 3:**
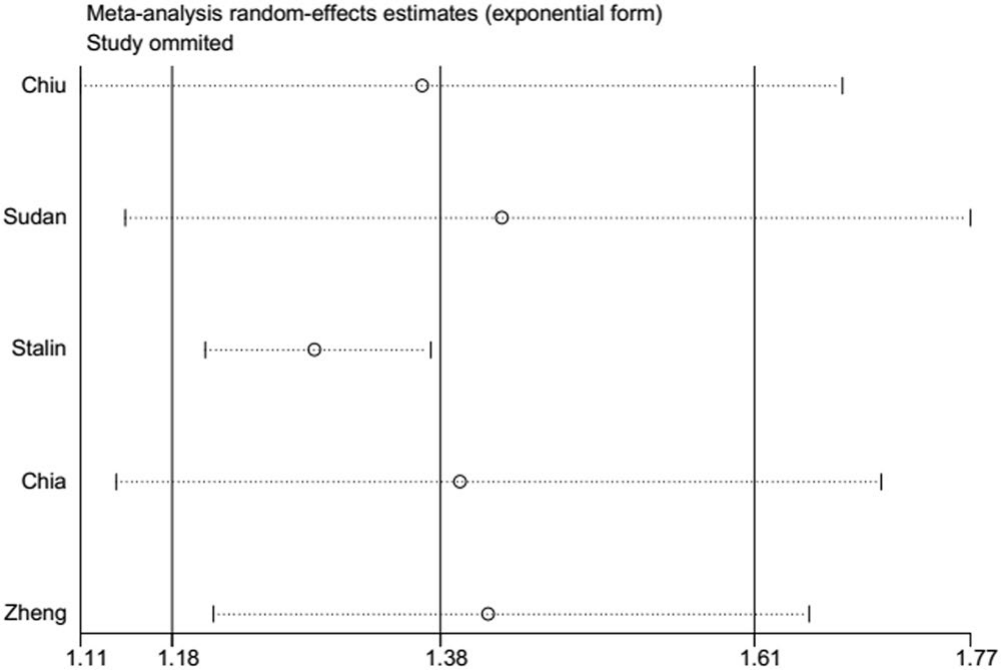
Plot of sensitivity analysis by excluding one study each time and the pooling estimate for the rest of the studies.

Among the seven included studies, five assessed the association between MP use or not and headache^18–21,24^. The pooled OR for association between MP use or not and headache are shown in Fig. 2. The combined result revealed higher risk of headache for MP user compared with non-MP user (OR, 1.38; 95% CI, 1.18–1.61) (*P* < 0.001, I^2^ = 58.9%).A sensitivity analysis was also performed by excluding one study each time and recalculating the pooled OR for the remaining studies and these data appear in Fig. 3. Visual inspection of the funnel plot did not show evidence of a significant publication bias (Supplementary Figure S1). Begg’s (*P* = 0.806) and Egger’s regression tests (*P* = 0.683) indicated no publication bias in this meta-analysis.

#### Long versus short MP call duration

**Figure 4:**
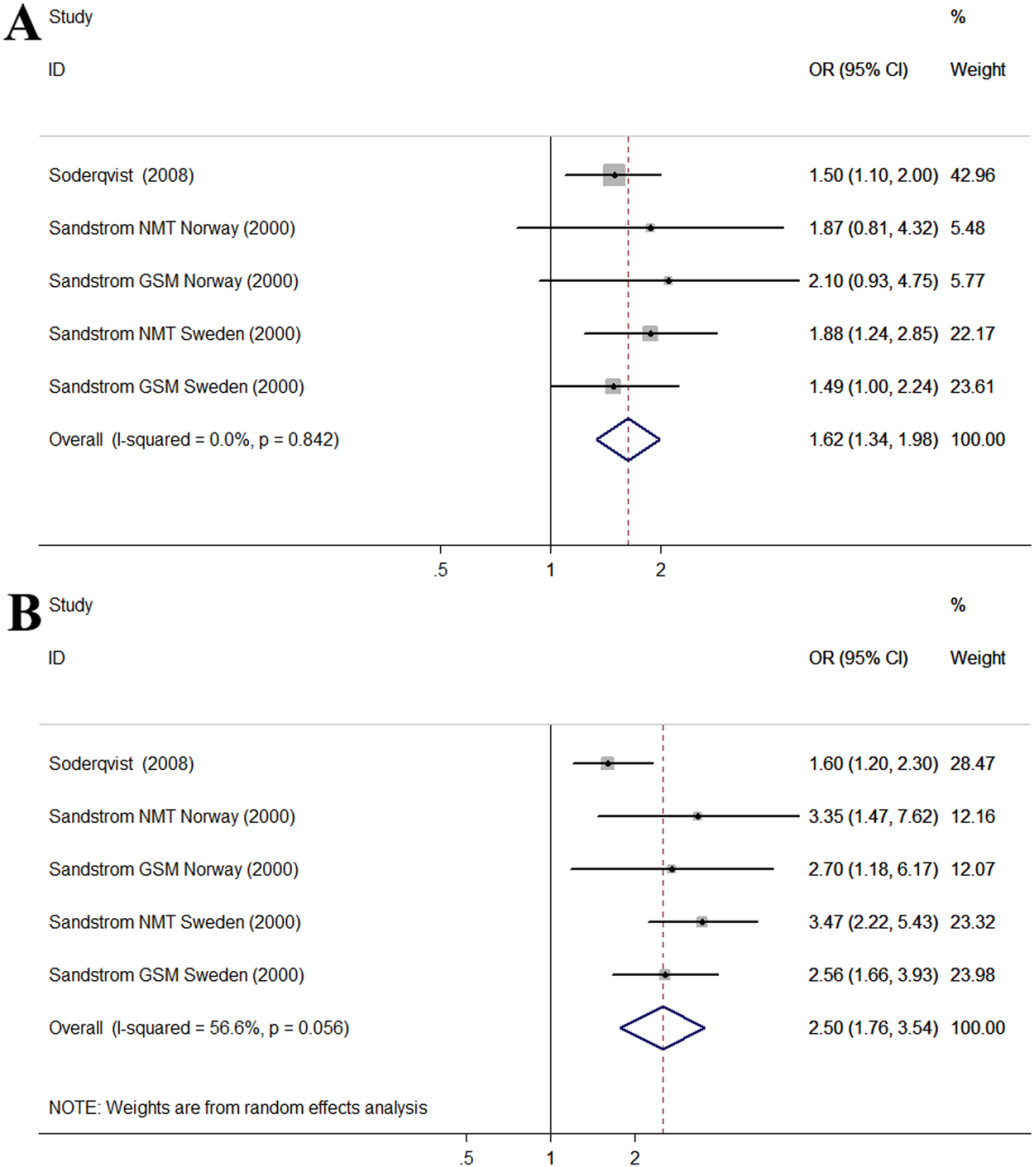
Forest plot of the association between different MP call duration and headache. (**A**) Forest plot for 2–15 min group compared with <2 min group; (**B**) Forest plot for >15 min group compared with <2 min group.

Two studies assessed the association between MP call duration and headache. According to the daily MP calling time, MP users were divided into different groups (<2 min group, 2–15 min group, >15 min group) and these data appear in Fig. 4. Compared with the <2 min group, the pooled OR was 1.62 (95% CI, 1.34–1.98) (*P* < 0.001, I^2^ = 0.0%) for 2–15 min group and 2.50 (95% CI, 1.76–3.54) (*P* < 0.001, I^2^ = 56.6%) for >15 min group. The result showed an increased risk of headache in those who had longer daily MP calling time.

#### High versus low MP use frequency

**Figure 5:**
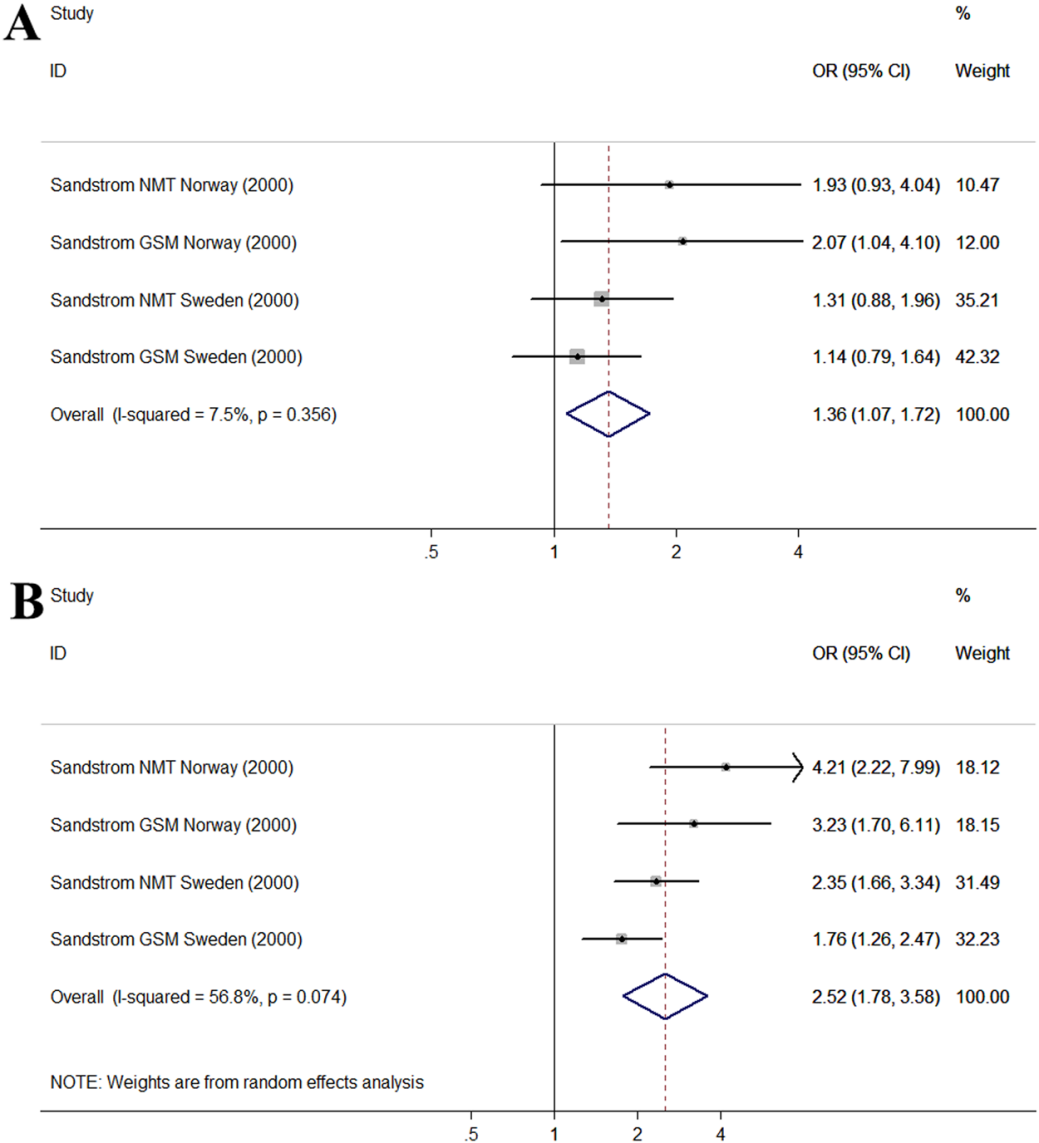
Forest plot of the association between different MP call frequency and headache. (**A**) Forest plot for 2–4 calls group compared with <2 calls group; (**B**) Forest plot for >4 calls group compared with <2 calls group.

Only one study investigated the association between the MP use frequency and headache but the study was performed in two countries and the author respectively assessed the association between digital and analog system MP use frequency and headache. This created 4 study groups and these data appear in Fig. 5. According to the daily MP use frequency, MP users were divided into different groups (<2 calls group, 2–4 calls group, >4 calls group). Compared with the <2 calls group, the pooled OR was 1.37 (95% CI, 1.07–1.76) (*P* < 0.001, I^2^ = 7.5%) for 2–4 calls group and 2.52 (95% CI, 1.78–3.58) (*P* < 0.001, I^2^ = 56.8%) for >4 calls group. The results showed an increased risk of headache in those who had higher daily MP use frequency.

## Discussion

To our knowledge, no meta-analysis has examined the relationship between MP use and headache. Our study, based on 7 available cross-sectional studies, first quantified the association of MP use with headache and we noted that MP users had increased risk of headache compared with non-MP users. Among MP users, the risk of headache was also increased in those who had longer daily call duration and higher daily call frequency. This finding substantiates the data demonstrating an association between MP use and an increased risk of headache and emphasizes the daily call duration and frequency as important influencing factors.

Studies to investigate any association between MP use and headache have used different methodologies. The pooled results of our meta-analysis were consistent with most studies and evidence of a significant association between MP use and headache was noted. Studies excluded from our meta-analysis suggested that increased risk of headache was correlated with MP use. Consistent with our results, Redmayne’s group^25^ found the number and duration of cellphone calls were significantly associated with headache (>6 cellphone calls >10 min weekly, adjusted OR 2.4, 95% CI 1.2–4.8). Szyjkowska and colleagues found that the OR for headache related to MP use longer than 30 min daily was 18.8^26^. In Khan’s study, the percent of chronic headache was related to daily MP use duration (5.03% in ≤30 min group, 24.69% in 30–60 min group, 39.39% in 60–90 min group and 30.76% in >90 min group, *p* < 0.0001)^27^. Moreover, in a large national cohort study of 420,095 Danish people, Schuz and colleagues found that standardized hospitalization ratios were increased by 10–30% for migraine in MP users corresponding to time since first subscription to a MP^28^. In addition, some other studies not included in our meta-analysis also indicated the possible association between MP use and headache^29–32^.

The underlying mechanism of the association between MP use and headache remains unclear but some suggest that breakdown of the blood-brain barrier due to exposure to low intensity MP frequency microwave energy may be involved^33–36^. Also, the dopamine-opiate system may be involved in headaches and low intensity electromagnetic energy exposure affects those systems^37–39^. However, since Frey’s group first reported headaches occurring after microwave energy exposure at approximately the same frequencies and incident energies that present day MP emit^40^, the exact mechanism under this association is still not fully understood now.

The results of our meta-analysis and lots of previous studies herein supported current clinical opinion that MP use may cause increased risk for headache. Therefore, it is advisable to admit that the use of MP is a risk factor for headache. In Stalin’s study^18^ and Chiu’s study^19^, the prevalence of MP usage among adult and children was 69.8% and 63.2% respectively in their study population, and that was only the data from two years ago. We could foresee the prevalence of MP usage will be higher in the future. So it is also advisable to suggest that excessive use of MP should be avoided by increasing social awareness through health promotion activities. It is imperative that health care professionals, clinicians and common people are educated about the deleterious influence of MP on headache. And it is reasonable to instruct children and adolescent about a prudent use of MPs. In addition, we encourage screening of headache patients during routine clinical visits to identify those patients to explore excessive MP use as a potential cause. Intervention and policies must be developed, evaluated and carry out at the population level to raise the awareness of the potential adverse health effect to decrease the headache caused by MP using.

The strengths and limitations of our study should be carefully considered. We have reported a comprehensive meta-analysis based on 7 cross-sectional studies and assessed the association between MP use and headache. And the studies included were all moderate-high quality. Our study only included studies used multiple logistic regression to decrease the effect of confounders for the MP use-headache association. However, our study was limited by the small number of studies available for combining. Meanwhile, all included studies are from Asia and Europe and the age of study population of included studies was not exactly the same. And this may introduce some heterogeneity when the results were combined. More large and high qualitative epidemiologic studies are required to further assess the association between MP use and headache. In addition, further experimental research is needed to explore more on the mechanism of this association.

In conclusion, our meta-analysis suggested that MP use is significantly associated with headache. More experimental and epidemiologic studies are still required to further affirm and understand this association.

## Electronic supplementary material

Supplementary Material
