# Supplementary material for: Mobile Phone Use and The Risk of Headache: A Systematic Review and Meta-analysis of Cross-sectional Studies
Source: Sci Rep. 2017 Oct 3;7:12595. doi: 10.1038/s41598-017-12802-9 (PMC5626766; doi:10.1038/s41598-017-12802-9)
Supplement: Supplementary file 1 — Supplementary Material [file 41598_2017_12802_MOESM1_ESM.pdf]

Mobile Phone Use and The Risk of Headache: A Systematic Review and Meta-analysis of Cross-sectional Studies

Jing Wang[1,2], Hui Su[1],Wei Xie[1],Shengyuan Yu[1*]

1. Department of Neurology, Chinese PLA General Hospital, Beijing 100853, PR China
2. School of Medicine, Nankai University, Tianjin 300071, PR China

*Corresponding author: Shengyuan Yu
Department of Neurology, Chinese PLA General Hospital, Beijing 100853, PR China
E-mail: yusy1963@126.com
Tel: +86-135-0117-1068

Table S1 Characteristics of literatures included in the meta-analysis

Study	Study design	Study population	Study size	Age	Gender	Exposure source	Study groups	Outcome assessment	Confounder adjusted	Study quality
Stalin 2016 [18]	cross-sectional	Adult of Southern India	2054	>18	Men and women	Mobile phones	Mobile phone user, Non-mobile phone user	Headache	Age, sex and socio-economic status	High (7 stars)
Chiu 2015 [19]	cross-sectional	Children of 40 districts in 25 cities/ counties of Taiwan	2042	11-15	Boys and girls	Mobile phones	Mobile phone user, Non-mobile phone user	Headache and migraine	grade, sex, residential area, type of school, guardian's educational level, and density of high-voltage transmission lines	Moderate (6 stars)
Zheng 2014 [20]	cross-sectional	Children in Chongqing city of China	746	9-12	Boys and girls	Mobile phones	Mobile phone use years (0 year, 1 year, >1 year)	Headache	sex, age, rural/urban residence, academic stress, daily exercise and having a recent cold or flu	Moderate (5 stars)
Sudan 2012 [21]	cross-sectional	Children registered at Danish National	8213	7	Boys and girls	Cell phones	Exposure to cell phones, no exposure to cell phones	Headache related symptoms, migraine	child's sex, mother diagnosed with migraines, mother's feelings	Moderate (6 stars)

		Birth Cohort							of worry, burden, and stress during pregnancy, mother’s age, social-occupational status, and child’s exposure to environmental tobacco smoke in the home	
Söderqvist 2008 [22]	cross-sectional	Swedish adolescents	1269	15-19	Boys and girls	digital 3G phones, GSM-phones, NMT-phones	Regular mobile phone use(≥2 min/day), non-Regular mobile phone use(<2 min/day)	Headache	Age and gender	Moderate (6 stars)
Sandström 2001 [23]	cross-sectional	company subscribers from Sweden and the southern part of the Norway	4504 in Sweden 1869 in Norway	>18	Men and women	GSM-phones, NMT-phones	Calling time per day (<2min, 2-15min, 15-60min, >60 min)	Headache	age, gender, geographical location of workplace, amount of video display terminal (VDT) work, occupation and	High (7 stars)

Chia 2000 [24]	cross-sectional	Residents of northeastern sector of Singapore	808	12-70	Men and women	Hand phones (HP)	HP user, non-HP user	Headache	psychosocial factors<br>age, sex, ethnic group, use of video display terminals, and occupational group	Moderate (6 stars)

Table S2 Characteristics of literatures investigating the association between mobile phone use and headache but not included in the meta-analysis

Study	Study design	Study population	Study size	Age	Gender	Exposure source	Study groups	Outcome assessment	Main Finding
Redmayne 2013 [25]	Cross-sectional	Adolescents of the Wellington Region, New Zealand	373	10-13	Boys and girls	Cell phone	Frequency of cell phone calls >10 minutes weekly (0,1-6,7-35)	Headache	>6 cell phone calls over 10 minutes weekly, adjusted OR:2.4, 95%CI:1.2-4.8
Szyjkowska 2014 [26]	Cross-sectional	Mobile phone subscriber in urban area of 4 regions of Poland	587	33±11	Men and women	Mobile phones	Daily Mobile use duration (≤30min, >30min; ≤15min, >15min)	Headache	Longer than for 30 min daily: OR:18.8, p <0.001<br>Longer than for 15 min daily: OR:9.6, 95%CI:1.91-49.05
Khan 2008 [27]	Health survey	Second year medical students	330	>18	Men and women	Mobile phones	Daily Mobile use duration (≤30min, 30-60min, 60-90min, ≥90min)	Chronic Headache	Percentage of headache: 5.03% in <30 min group, 24.69% in 30-60 min group, 39.39% in 60–90 min group and 30.76% in >90 min group, p <0.0001
Schuz 2009 [28]	Cohort study	Mobile phone	420095	>18	Men and women	Mobile phones	Mobile phone use years (0	Migraine	SHRs for migraine:<br>1 year:

		subscriber in Denmark					year, 1 year, 1-4 years, 5-9 years and ≥10years)		SHR:1.3,95%CI:1.1-1.5<br>1-4 years: SHR:1.2,95%CI:1.2-1.3<br>5-9 years: SHR:1.2,95%CI:1.1-1.3<br>≥10 years: SHR:1.1,95%CI:0.8-1.4<br>Total: SHR:1.2,95%CI:1.1-1.3
Cho 2016 [29]	Cross-sectional	Participants of the KoGES-Ansan	532	>40	Men and women	Mobile phones	Daily Mobile use frequency and duration (Average frequency of calls/day: <5 calls, 5-10 calls, ≥10calls; Average call duration/call: <5 min, 5-10 min, ≥10 min)	Headache (HIT-6 score)	Average frequency of call per day correlates with HIT-6 (male, r=0.241)<br>Average duration of one call correlates with HIT-6 (male, r=0.227; female=0.191)<br>Mean HIT-6 score: 42.48 in <5min group, 45.98 in ≥5 min group, p <0.001<br>Compared with <5min group, OR was 2.22,95%CI:1.78-2.49 for moderate headache in ≥5 min group, OR

									was 4.44,95%CI:2.11-8.90 for severe headache in ≥5 min group
Balikci 2005 [30]	Cross-sectional	Residents randomly selected from Elazig, east part of Turkey	695	NA	Men and women	Mobile phones	Mobile phone use years (0 year, 1 year, 2 years, 3 years and ≥4 years)	Headache	Among people have headache symptom: 27.9% have headache before mobile phone use and 72.1% have headache after mobile phone use.<br>Headache percentage: Non-mobile phone user 63.0%,<br>Total mobile phone user 78.9%, $p < 0.01$
Küçer 2014 [31]	Cross-sectional	Residents randomly selected from Turkey	350	9-80	Men and women	Mobile phones	Daily Mobile use duration (≤16min, >16min)	Headache	Headache percentage:<br>≤16min group: 20.9%<br>>16min group: 40.3%<br>$p < 0.05$
Mortazavi 2007 [32]	Cross-sectional	Students of 2 university in Iran	518	NA	Men and women	Mobile phones	Not mentioned	Headache	Headache (53.5%)was the most common self-reported symptom, but no association was found between mobile

phone use and headache

KoGES, Korean Genome and Epidemiology Study; NA, not available; HIT-6, Headache Impact Test-6

Table S3 Assessment of cross-sectional studies included in this meta-analysis

First author, year	Representativeness of the sample	Sample size	Non-respondents	Ascertainment of the exposure (risk factor)	Comparability	Assessment of the outcome	Statistical test	Total score
Chiu 2015	-	*	-	*	**	*	*	6 stars
Zheng 2014	-	-	-	*	**	*	*	5 stars
Sudan 2012	-	*	-	*	**	*	*	6 stars
Stalin 2016	*	*	-	*	**	*	*	7 stars
Chia 2000	*	-	-	*	**	*	*	6 stars
Söderqvist 2008	-	*	-	*	**	*	*	6 stars
Sandström 2001	*	*	-	*	**	*	*	7 stars

** quality criterion completely satisfied; * quality criterion satisfied; - quality criterion not satisfied or insufficient information to adjudicate as satisfied

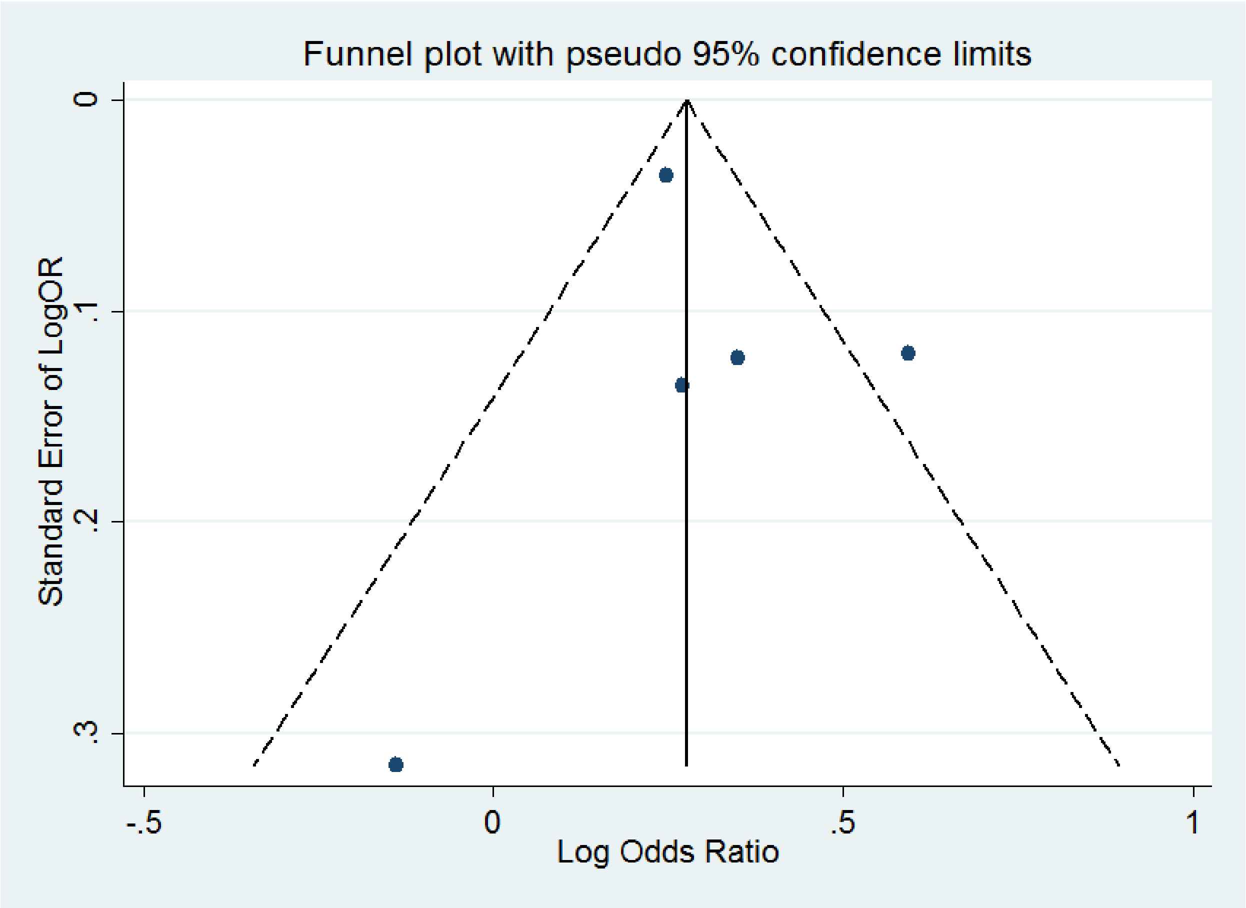

Fig. S 1 Funnel plot of log relative risk vs. standard error of log relative risks for MP user vs. non-MP user
